# First episode of chronic anterior uveitis in patients with juvenile idiopathic arthritis and relationship with administrated treatments

**DOI:** 10.1186/1546-0096-12-S1-P119

**Published:** 2014-09-17

**Authors:** Alina Lucica Boteanu, Carmen Velazquez Arce, Carlos Guillen Astete, Maria Angeles Blazquez Cañamero, Maria Luz Gámir Gámir

**Affiliations:** 1 Rheumatology, University Hospital Ramon y Cajal, Madrid, Spain

## Introduction

Chronic anterior uveitis is one of the most serious complications of juvenile idiopathic arthritis (JIA), showing an increased incidence in patients with early-onset disease, oligoarticualar, ANA (+). Biologic therapy used in the treatment of JIA has greatly improved the articular prognosis, however their effectiveness in JIA associated uveitis is still not well established, having reported new cases of uveitis during treatment with anti -TNF, especially during treatment with etanercept.

## Objectives

The objective of this work is to report the incidence of new cases of chronic anterior uveitis in patients who were treated with Methotrexate (MTX), Adalimumab (ADM) or Etanercept (ETN) for JIA.

## Methods

We performed a retrospective observational study of 70 patients with diagnosis of early-onset JIA with high risk of developing chronic anterior uveitis. There were recorded the new episodes of uveitis per patient. Incidence was calculated as the number of new episodes per 100 patients per year of follow-up according to the ILAR category and treatment and based exclusively on the treatment. Comparisons were made using T Student test.

## Results

Fifty-six (80.0%) patients were female and 14 (20.0%) males. The categories ILAR were: 42 (60.0%) oligoarticular, 10 (14.3%) extended oligoarticular, 15 (21.4%) poliarticular and 3 (4.3%) associated with psoriasis. The average age at diagnosis was 3.5 SD 1.6 years (median 3 years). ANA positive were found in 41 (58.6%) patients. The mean follow-up of patients was 4.44 SD 3.65 years. There were 5 new cases of uveitis during treatment (3 MTX, 2 MTX-ETN or ETN and 0 with ADM only) and 10 cases presented to debut or before starting DMARDs or biologic therapy. The incidence was 0.15 and 0.11 cases per 100 patient / year, respectively (p> 0.05, t Student test). The accompanying table describes the distribution of new cases according to the ILAR category and administered treatment during the follow up period.

## Conclusion

The incidence of occurrence of uveitis in our series of JIA patients at high risk of uveitis is comparable to that described in the literature (22.8%) and similar between patients who received MTX as monotherapy and those who received MTX and ETN or ETN. We reported no new cases of uveitis in patients treated with ADM, although in 85% of cases ADA was recomended in patients diagnosed with uveitis, with studies that have shown that it is more effective than treating this complication with ETN. The number of cases that occurred during treatment with ETN in our series of cases is too small to infer a causal relationship, the stratification by immunological profile, sick time, age or baseline severity of the symptoms could influence the development of this complication. Prospective comparative studies of biological and non-biological DMARDs are needed to assess the increase or decrease in the risk of uveitis associated with JIA.

## Disclosure of interest

None declared.

